# Neuropeptides SP and CGRP Underlie the Electrical Properties of Acupoints

**DOI:** 10.3389/fnins.2018.00907

**Published:** 2018-12-12

**Authors:** Yu Fan, Do-Hee Kim, Yeonhee Ryu, Suchan Chang, Bong Hyo Lee, Chae Ha Yang, Hee Young Kim

**Affiliations:** ^1^Department of Physiology, College of Korean Medicine, Daegu Haany University, Daegu, South Korea; ^2^Korean Medicine Fundamental Research Division, Korea Institute of Oriental Medicine, Daejeon, South Korea

**Keywords:** substance P, CGRP, acupoints, electrical properties, skin conductance, Evans blue dye, plasma extravasation, neurogenic inflammation

## Abstract

Electrical skin measurements at acupuncture points (acupoints) have been utilized as a diagnostic and therapeutic aid for more than 50 years. Although acupoints are described as having distinct electrical properties, such as high conductance and low impedance, the underlying mechanisms are currently unknown. The present study investigated in a rat model of hypertension whether the high conductance at acupoints is a result of the release of the neuropeptides substance P (SP) and calcitonin gene-related peptide (CGRP) during neurogenic inflammation in the referred pain area. When plasma extravasation from neurogenic inflammation was examined by exploring the leakage of intravenously injected Evans blue dye (EBD) to the skin, extravasated EBD was found most frequently in acupoints on the wrist. The increased conductance and temperature at these acupoints occurred during the development of hypertension. The increase in conductance and plasma extravasation at acupoints in hypertensive rats was ablated by cutting median and ulnar nerves, blocking small diameter afferent fibers with resiniferatoxin (RTX) injection into median and ulnar nerves, or antagonizing SP or CGRP receptors in acupoints. In turn, intradermal injection of SP or CGRP resulted in increased conductance and plasma extravasation in naïve rats. Elevated levels of SP and CGRP were found in the acupoints of hypertensive rats. These findings suggest that the high conductance at acupoints is due to vascular leakage following local release of SP and CGRP during neurogenic inflammation.

## Significance Statement

Electrical skin measurements at acupuncture points have been utilized as a diagnostic and therapeutic aid for more than 50 years. Although acupoints are described as having distinct electrical properties, such as higher conductance and lower impedance than that of surrounding skin, the underlying mechanisms are completely unknown. Using a newly constructed electrode, intravenous injection of Evans blue dye, and cutaneous thermal recordings and imaging, the present study suggests a novel mechanism underlying the electrical properties of acupoints: the neuropeptides SP and CGRP produce high conductance at acupoints by causing neurogenic inflammation, plasma extravasation and accumulation of subskin water contents.

## Introduction

Acupuncture, a therapeutic intervention of traditional medicine, has been used for centuries to relieve a variety of conditions. For acupuncture treatment, thin needles are inserted into specific but poorly defined sites on or under the skin called acupoints or acupuncture points. Based on acupuncture theory, there are about 360 acupuncture points, most of which lie along the Qi channels (called meridians) connecting the surface of the body to internal organs. Each acupoint communicates with a specific internal organ; an acupoint reflects the status of an internal organ, and the internal disorders can be treated by stimulating the acupoints (Stux and Pomeranz, 2012). In support of this, we and others have proven that acupoints become hypersensitive under abnormal visceral conditions and that stimulation of the acupoints can relieve the symptoms of the associated visceral organs (Chae et al., 2007; Kim et al., 2017).

As the acupoints themselves are grossly anatomically invisible, several scientific approaches, such as electrodermal measurements (Ahn et al., 2008) and infrared thermal imaging (Yang et al., 2007), have been attempted to identify the acupoints. Notably, numerous studies have reported the electrical properties of acupoints. Since the 1950s, when Nakatani (1956) reported that there were some points on the skin with special electrical properties, experimental and clinical studies have been carried out in many countries including China, Japan, France, Germany, and the United States and suggest that acupoints have distinct electrical properties, including a higher conductance, lower impedance and resistance and increased capacitance compared to the surrounding skin (Ahn et al., 2008). As this view gained traction, many instruments such as acupoint detectors and electrodiagnostic devices have been developed and are increasingly used in acupuncture clinics. However, the mechanisms by which acupoints have these distinct electrical properties are currently unknown.

Our previous studies showed that in rat models of hypertension or colitis, the skin over acupoints exhibits neurogenic inflammation due to viscerosomatic convergence in sensory pathways (Kim et al., 2006, 2017). Neurogenic inflammation is characterized by vasodilation and vascular leakage (plasma extravasation) in the skin arising from the release of neuropeptides calcitonin gene-related peptide (CGRP) and substance P (SP) from activated small diameter sensory afferents (Schmelz and Petersen, 2001). The insights that many acupoints show neurogenic inflammation under certain conditions (Kim et al., 2006, 2017) and electrically high conductance/low impedance (Ahn et al., 2008; Colbert et al., 2008, 2009) have led to the hypothesis that the neuropeptides CGRP and SP evoke vascular dilation and leakage, causing the increased subskin tissue water content in acupoints and thus producing electrically high conductance and low impedance, potentially underlying the electrical properties of acupoints. To prove this hypothesis, the present study used a rat model of immobilization-induced hypertension (IMH) to investigate (1) whether acupoints exhibit active neurogenic inflammatory responses by using intravenous injection of Evans blue dye (EBD) and cutaneous thermal recordings and imaging and (2) whether acupoints have high conductance by using a newly constructed electrode and plasma extravasation. Furthermore, we explored (3) whether the increased conductance at acupoints is mediated by activation of small diameter afferents and generated by localized release of CGRP and SP.

## Materials and Methods

### Animals

Adult male Sprague-Dawley rats (Hyochang, Seoul, South Korea) weighing 250–350 g were used. Animals were housed at constant humidity (40∼60%) and temperature (22 ± 2°C) on a 12-h light/dark cycle and allowed free access to food and water. All procedures were carried out in accordance with the National Institutes of Health Guide for Care and Use of Laboratory Animals and approved by the Institutional Animal Care and Use Committee (IACUC) at Daegu Haany University.

### Chemicals

Evans blue dye (50 mg/ml saline; Sigma-Aldrich, St. Louis, MO, United States), human calcitonin gene-related peptide (CGRP; 1 mg/ml saline; Bachem, Torrance, CA, United States; a 37-amino acid peptide), and α-CGRP 8-37 (1 mg/ml distilled water, Bachem, Torrance, CA, United States; a CGRP receptor antagonist) were used in this study (Shen et al., 2001). Substance P acetate salt hydrate (SP; 0.5 mg/ml saline; Sigma-Aldrich; a SP receptor agonist) and (+)-(2S,3S)-3-(2-methoxybenzylamino)-2-phenylpiperidine (CP-99994; 33 mmol/ml saline; Sigma-Aldrich; an SP receptor antagonist) were also used (McLean et al., 1993). Resiniferatoxin (RTX; 100 μg/ml vehicle; Sigma-Aldrich), an ultrapotent capsaicin analog known to block small diameter afferent fibers containing transient receptor potential vanilloid type 1 receptor (TRPV1) (Suter et al., 2009), was dissolved in a vehicle that contained 0.3% Tween 80, 10% DMSO and saline. Capsaicin (0.05 and 0.1%; Sigma-Aldrich; a TRPV1 agonist) was dissolved in a vehicle consisting of 10% alcohol and 10% Tween 80 in saline (Knotkova et al., 2008), and FITC-conjugated isolectin B4 (FITC IB4 tracer; 1%, 3 μl, Vector Laboratories, Burlingame, CA, United States) was used.

### Immobilization Stress-Induced Hypertension (IMH) and Measurement of Systolic Blood Pressure

Hypertension was induced by immobilization with a cone-shaped polyethylene bag, as described previously (Kvetnansky et al., 1979). Systolic blood pressure was measured non-invasively with a tail cuff blood pressure monitor (Model 47, IITC, Inc., Woodland Hills, CA, United States). Briefly, the rat was placed in a chamber, and an occluding cuff and a pneumatic pulse transducer were positioned on the base of the tail. A programmed electrosphygmomanometer (Narco Bio-Systems, Inc., Austin, TX, United States) was inflated and deflated automatically, and the tail cuff signals from the transducer were automatically collected every 10 min using an IITC apparatus (Model 47, IITC, Inc.). The mean of two readings was taken at each blood pressure measurement.

### Detection of Neurogenic Inflammation in the Skin by EBD Injection

Neurogenic inflammatory sites were visualized by intravenously injecting EBD (50 mg/kg) in male Sprague-Dawley rats as described previously (Kim et al., 2017). While the rats were immobilized by the cone-shaped bags, the distal portion of the tail was dipped into 40°C warm water for at least 30 s. EBD was then injected into the tail vein with a catheter (26 gauge), and skin color changes were observed up to 2 h after the injection. The blue-dyed areas on the skin were sketched using body charts, photographed and compared with a human acupoint chart based on the transpositional method, which locates acupoints on the surface of animal skin corresponding to the anatomic site of human acupoints (Yin et al., 2008).

### Measurement of Skin Surface Temperature

Skin temperature was measured using a K-type thermocouple microprobe (TC-11p, Minnesota Measurement Instruments, Minneapolis, MN, United States) coupled with an analog-digital interface converter (Physitemp BAT-12, American Laboratory Trading, San Diego, CA, United States) and digitized through a PowerLab 4/30 acquisition system (ADInstruments, Colorado Springs, CO, United States). While the rats were placed in a plastic chamber, a flexible thermoprobe was attached to the skin over wrist acupoints, mostly PC6 acupoints, the nearby site approximately 5 mm away from PC6 (Figures 2A,B) or capsaicin-injected sites (Figure 2C). Body temperature was also monitored with a regular thermocouple probe inserted into the rectum. For the experiment shown in Figures 2A,B, after the basal temperatures were recorded for at least 10 min, the rats were subjected to the IMH procedure, and the temperatures were measured up to 30 min after restraint. The skin-body temperature differentials were estimated by subtracting the body temperature from the skin temperature at each time point. At 30 min after restraint, infrared thermal images were obtained using a thermal camera (Ti55, Fluke IR Fusion Technology, Washington DC, United States) under slight isoflurane anesthesia. In another set of experiments (for Figure 2C), either capsaicin (0.05 or 0.1% capsaicin, 30 μl) or vehicle was intradermally injected into the bilateral PC6 acupoint near the wrist under slight isoflurane anesthesia, and temperature changes in the injected sites were monitored up to 30 min after injection.

### Measurement of Electrical Skin Conductance

Skin moisture and temperature in the rats may be influenced according to external environment. To control the factors, all experiments were carried out under constant humidity (40∼60%) and temperature (22 ± 2°C). The animal hair was shaved prior to the placement of electrode. To simultaneously measure conductance and the applied pressure, a device was newly constructed by coupling a force transducer (FT-100, iWorx/CB Sciences, Inc., Dover, NH, United States) with an electrical conductance probe (3.7 mm diameter, stainless). The rats were placed in cone-shaped bags or plastic chambers. While the positive electrode was attached to the tail surface, the device (negative electrode) was placed on the skin over acupoints and was pressed at a force of 300 g. Signals from the conductance (current) probe and the force transducer were fed to an ETH-200 Bridge Amplifier (CB Science, Inc., Lemont, PA, United States) and a GSR AMP device (Model FE116, ADInstruments, Colorado Springs, CO, United States), respectively, and digitized through a PowerLab 4/30 acquisition system (ADInstruments). After basal electrical currents were recorded for at least 5 min, skin conductance (measured as a current) following treatments was recorded up to 30 min.

### Estimation of EBD Extravasation in Skin Tissues

To assess the extent of EBD extravasation in the skin, rats were sacrificed 120 min after intravenous injection of EBD. The skin near the wrist was photographed using a digital camera (SONY ILCE-5000, China). The original EBD images were used to obtain a three-dimensional (3D) surface plot with ImageJ software (National Institute of Mental Health, Rockville, MD, United States) using the command Plugins/3D/interactive 3D surface plot (parameters; Mesh, Spectrum LUT, Scale 1.75, and Z 0.19). To determine the concentration of EBD that infiltrated the skin tissues, the skin tissue was processed using the dye-extraction method described previously (Martin et al., 2010). In brief, the skin (approximately 3 mm in size) near the wrist was excised, dry-weighed, homogenized in 1: 3 volume of 50% trichloroacetic acid (TCA; dissolved in 0.9% saline) and centrifuged (10,000 rpm for 10 min). The supernatants were diluted with 1: 3 volume of 95% ethanol and measured using a spectrophotometric method (620 nm excitation/680 nm emission).

### Surgical or Pharmacological Blockade of Median and Ulnar Nerves

Median and ulnar nerves were blocked surgically or pharmacologically as described previously (Kim et al., 2013). Briefly, under isoflurane anesthesia, a small skin incision was made longitudinally on the medial part of the elbow to expose the median and ulnar nerves. For surgical lesions of median and ulnar nerves, the nerves were bilaterally ligated with 4–0 silk and cut around the medial head of the triceps muscle of both forelimbs. The sham group underwent the same procedure but without nerve injury. For blocking small diameter afferent fibers, RTX (0.01%, 30 μl) was administered perineurally in the median and ulnar nerves. All incisions were closed aseptically and 2 or 3 days after surgery, experiments for skin conductance were performed.

Another set of experiments was performed to confirm the blockade of small diameter afferent fibers by RTX by using an IB4 tracing method (Pan et al., 2003). In brief, 48 h after perineural injection of either RTX (*n* = 3) or vehicle (*n* = 3) into the ulnar nerve, an FITC IB4 tracer (3 μl) was administered into ulnar nerves under isoflurane anesthesia in rats (*n* = 3). Three to four days later, dorsal root ganglion (DRG) neurons of C8 and T1 were removed, post-fixed in 4% buffered paraformaldehyde (PFA) for 2 h, immersed in 30% sucrose overnight and cryosectioned at 30 μm. The cryosections were then mounted on gelatin-coated glass slides. Skin images were taken from 3 to 4 sections per animal under an Olympus AX70 fluorescence microscope (Olympus, Japan) and quantified by using ImageJ software.

### Immunohistochemistry for CGRP or SP in the Skin

One hour after restraint, skin samples were taken from the wrist, which most commonly showed EBD leakage in IMH rats (*n* = 6) and naïve rats (*n* = 6). The skin samples were paraffin-embedded, sectioned (5 μm), and incubated with either anti-CGRP mouse antibody (1:500; Chemicon, Temecula, CA, United States; RRID:AB_1658411) or anti-SP mouse monoclonal antibody (1:500; GeneTex, Irvine, CA, United States; RRID:AB_785913), followed by incubation with secondary antibody (1:500, Alexa Fluor 488-conjugated donkey anti-mouse IgG antibody, Thermo Scientific, Waltham, MA, United States; RRID: AB_141607). The sections were mounted on gelatin-coated slides, air-dried, and coverslipped. Skin images were taken from three sections from each animal with a laser-scanning confocal microscope (LSM700, Carl Zeiss, Germany) and quantified by using ImageJ software (National Institute of Mental Health, Rockville, MD, United States). The number of pixels with green fluorescence intensity greater than the cut-off value (100) was counted to quantify positive staining. Data were expressed as the number of positive pixels over a field area of 1280 × 1024 pixels.

### Statistical Analysis

Statistical analysis was carried out using SigmaStat 3.5 software (Systat Software, Inc., United States). All data are presented as the mean ± SEM (standard error of the mean) and analyzed by one or two-way repeated measures analysis of variance (ANOVA) with Tukey *post hoc* tests or unpaired *t*-tests where appropriate. Statistical significance was considered at *p* < 0.05.

## Results

### Cutaneous Neurogenic Inflammation at Acupoints

**FIGURE 1:**
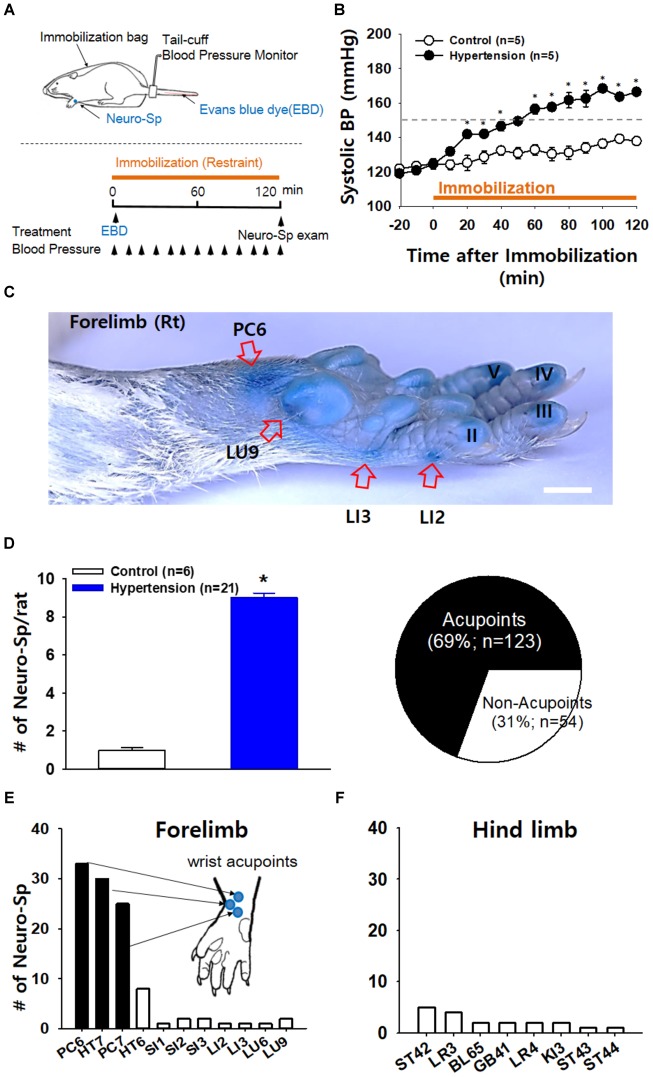
Neurogenic inflammation at acupoints in IMH rats. **(A)** Schematic of the experimental procedure in the rat model of immobilization-induced hypertension (IMH). Evans blue dye (EBD); neurogenic inflammatory spots [neurogenic spots (Neuro-Sp)]. **(B)** Development of hypertension following immobilization (restraint). In restrained rats, systolic blood pressure (systolic BP) was significantly elevated, reaching the level of hypertension over 150 mmHg within 1 h, compared to that in unrestrained control rats (control). ^∗^*p* < 0.001 vs. control. **(C)** A representative image of Neuro-Sp in an IMH rat. **(D)** Numbers of Neuro-Sp per animal (bar graph) and correlation of the anatomic location between Neuro-Sp and acupoints in hypertensive rats (*n* = 21 animals; pie graph). Numbers of Neuro-Sp corresponding to acupoints in the forelimb **(E)** or hind limb **(F)**. Bar = 20 mm.

First, we explored whether some of acupoints displayed neurogenic inflammation in IMH rats. When a rat was placed in an immobilization bag, systolic blood pressure gradually increased for the next several hours (Figures 1A,B), consistent with our previous study (Kim et al., 2017). Approximately 10 min after the initiation of restraint, cutaneous neurogenic inflammatory sites [neurogenic spots (Neuro-Sp)] were visualized by intravenous injection of EBD (50 mg/kg). The blue spots started to appear approximately 5 min after EBD injection, ranged in diameter from 0.5 to 3 mm and were maintained throughout the experiment. IMH rats exhibited approximately nine spots per animal (Figure 1D), while control rats only showed a few spots. When the Neuro-Sp in IMH rats (*n* = 21) were mapped and compared with the corresponding human anatomical acupoints, the majority appeared bilaterally or unilaterally on the wrist, and 69% (123 of 178 spots) were found in acupoints of the forelimbs, such as PC6 (33 spots), PC7 (25 spots), and HT7 (30 spots) (Figures 1D–F). These results indicate that acupoints, most frequently on the wrist, display cutaneous neurogenic inflammation and plasma extravasation in the rat model of IMH.

### Increased Skin Temperature at Acupoints Following Neurogenic Inflammation

**FIGURE 2:**
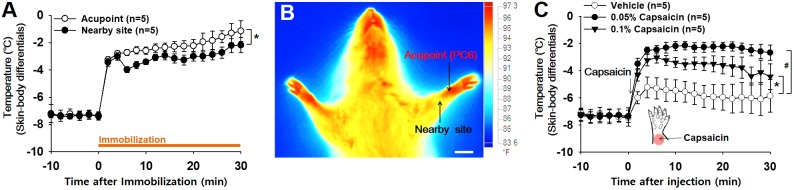
Increased temperature at acupoints in IMH rats. **(A)** Changes in temperature at acupoints on the wrist following restraint. Skin (acupoint)-body temperature differentials were calculated by subtracting the body temperatures from the temperatures over wrist acupoints. ^∗^*p* < 0.05 vs. nearby site. **(B)** A representative thermal infrared image of the wrist 2 h after restraint. **(C)** Effect of intradermal capsaicin on skin–body temperature differentials. Capsaicin (0.05 or 0.1%) or vehicle was injected into the skin over the wrist of naïve rats. ^∗^*p* < 0.05, 0.05% capsaicin vs. vehicle; ^#^*p* < 0.05, 0.1% capsaicin vs. vehicle. Bar = 20 mm.

To determine whether the acupoints undergo active neurogenic inflammation processes, cutaneous temperature, as an outcome measure of inflammation (Birklein and Schmelz, 2008; Montalto et al., 2013), was compared between acupoints on the wrist and nearby sites approximately 5 mm away from the acupoints in IMH rats. Skin temperature rapidly elevated following restraint and then continued to increase slowly over 30 min after restraint. This increase was higher at acupoints than at nearby sites (two-way ANOVA; group *F*_(1,4)_ = 26.231, *p* = 0.07; time *F*_(15,60)_ = 28.589, *p* < 0.001; interaction *F*_(15,60)_ = 1.061, *p* = 0.41; Figure 2A). Thermal infrared imaging found elevated temperature in the skin over wrist acupoints such as PC6 (Figure 2B). To verify whether neurogenic inflammation itself can increase skin temperature, the effect of intradermally injected capsaicin (0.05, 0.1%; 30 μl), which is known to trigger neurogenic inflammation (Lin et al., 1999), on skin temperature was investigated. Local injection of capsaicin into the skin of the wrist significantly increased temperature at the injection site in a dose-dependent manner compared to injection of vehicle (two-way ANOVA, group *F*_(2,8)_ = 29.018, *p* < 0.001; time *F*_(20,80)_ = 38.794, *p* < 0.001; interaction *F*_(40,160)_ = 9.737, *p* < 0.001; Figure 2C), suggesting that acupoints on the wrist of IMH rats are undergoing active processes of neurogenic inflammation.

### Increased Electrical Conductance and Plasma Extravasation at Acupoints in IMH Rats

**FIGURE 3:**
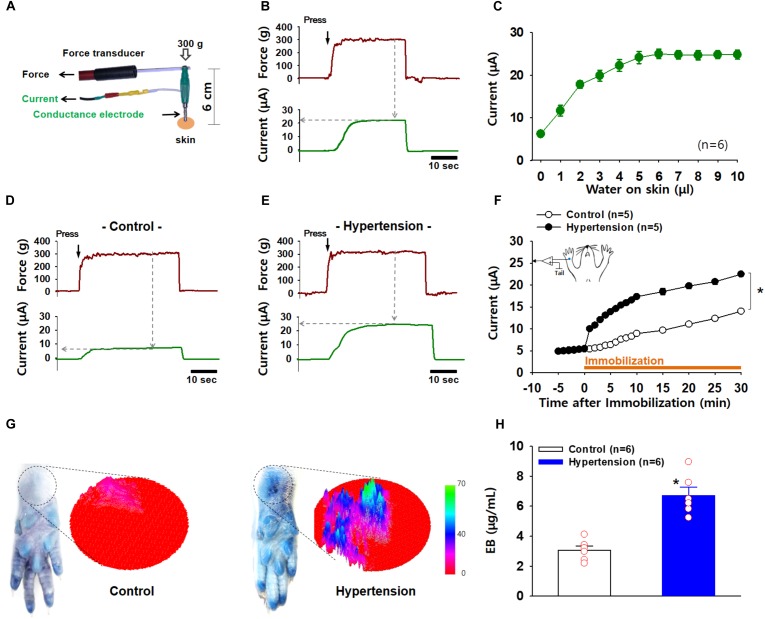
Increased electrical conductance and plasma extravasation at acupoints in IMH rats. **(A)** A newly constructed electrode for the simultaneous measurement of conductance and applied pressure. **(B)** A representative trace of applied force and electrical currents. Conductance was estimated as the maximum current value (μA) reached when a pressure of 300 g on the electrode was held constant. **(C)** Changes in conductance induced by various amounts of water topically applied to rat skin. **(D–F)** Increased conductance at acupoints following IMH. A representative trace of applied pressure (upper panels) and electrical currents (low panels) recorded in wrist acupoints of control and IMH rats. Significantly increased conductance at the wrist acupoint was observed in IMH rats compared to that in control **(F)**. *p* < 0.05 vs. control. **(G)** Extravasation of EBD over the wrist acupoint of hypertensive rats. Representative photographs of wrist areas in control and IMH rats 2 h after intravenous injection of EBD and the 3D images created from the circles in the photographs by using ImageJ. **(H)** Concentration of EBD in the wrist acupoints of naïve control and IMH rats. ^∗^*p* = 0.027 vs. control.

Next, we investigated whether acupoints showed increased electrical conductance under pathological conditions. The pressure exerted by the probe on the skin is considered to be a potential confounder affecting the reproducibility and reliability of data on the measurement of electrical skin conductance/impedance (Ahn and Martinsen, 2007). To hold the electrode to the skin at a constant pressure at every measurement, we constructed a conductance probe that could measure electrical currents and touch pressure simultaneously (Figure 3A). Skin conductance was estimated as the plateau value of electrical current recorded while holding the electrode at a constant pressure of 300 g (Figure 3B). To assess the validity of the device, various amounts of distilled water (1∼10 μl) were applied to the skin near the wrist in isoflurane-anesthetized rats (*n* = 6), and the electrical conductance was measured at the wrist skin. Electrical currents proportionally increased with increasing water volumes, reaching a maximum level at approximately 5 μl water (Figure 3C), indicating an increase in conductance according to the extent of skin hydration.

To explore whether acupoints exhibit a high conductance under pathological conditions, electrical currents at an acupoint on the wrist were compared between IMH and control rats. When the conductance probe was applied over the PC6 acupoint on the wrist at a constant pressure of 300 g, the acupoint in the IMH rats showed a higher electrical conductance than that in the control rats (two-way ANOVA; group *F*_(1,4)_ = 1147.149, *p* < 0.001; time *F*_(19,76)_ = 228.471, *p* < 0.001; interaction *F*_(19,76)_ = 232.043, *p* < 0.001; Figures 3D–F). In another set of experiments, we imaged and quantified neurogenic extravasation at acupoints on the wrist 2 h after EBD injection with or without restraint. Figure 3G shows that there was blue EBD staining over the wrist acupoints in IMH rats (hypertension) but not naïve control rats (control). The 3D plots derived from the photographs also show the predominant EBD staining in IMH rats (right panels in Figure 3G). When measured by spectrophotometry, the EBD concentration at the wrist acupoints was significantly higher in IMH rats (hypertension, *n* = 6) than in naïve control rats (control, *n* = 6; *p* < 0.001; Figure 3H). These results suggest that both conductance and plasma extravasation were enhanced at the acupoints of IMH rats.

### C-Fiber Mediation of the High Electrical Conductance of Acupoints

**FIGURE 4:**
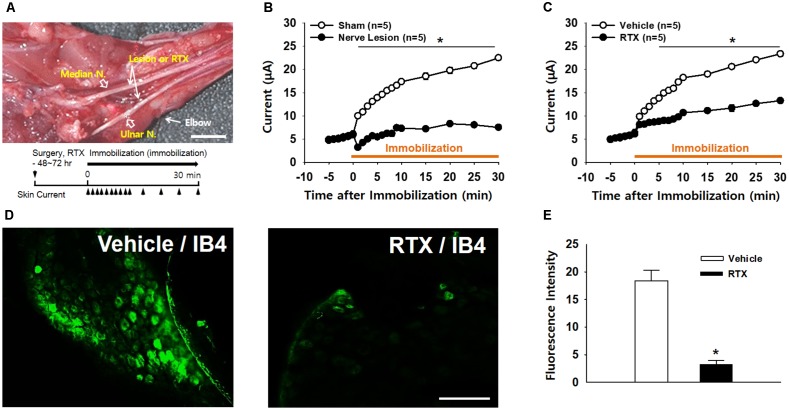
Blockade of high conductance at acupoints by either surgical nerve injury or perineural injection of RTX in IMH rats. **(A)** A photograph of the median and ulnar nerves at the plane of the elbow segment. Arrows indicate sites for surgical lesions or perineural injection of resiniferatoxin (RTX). Bar = 20 mm. **(B)** Effect of surgical nerve injury on the development of high conductance at acupoints in IMH rats. Peripheral nerve injuries abolished the development of high conductance at acupoints, compared to leaving the nerve intact (sham). ^∗^*p* < 0.05. **(C)** Effect of perineural injection of RTX on electrical skin conductance in IMH rats. **(D,E)** Epifluorescent images showing IB4-labeled neurons in the C8 DRG of a rat injected with IB4 into RTX- (RTX/IB4) and vehicle-treated (vehicle/IB4) ulnar nerves. An FITC IB4 tracer was bilaterally administered into nerves of rats 48 h after perineural injection of either RTX or vehicle into ulnar nerves. Significantly fewer IB4-labeled cells were found in the RTX-treated DRGs (*n* = 10 slices from three animals) than in those treated with vehicle (*n* = 10 slices from three animals, Vehicle). ^∗^*p* < 0.001 vs. vehicle. Bar = 100 μm.

To identify whether afferent nerves mediate the development of high conductance at acupoints, surgical lesions of the ulnar and median nerves were made 48∼72 h prior to restraint and skin conductance measurements (Figure 4A). While conductance at the wrist acupoint wrist gradually increased following restraint (sham; Figure 4B), such effects were not observed in the rats with nerve lesions (nerve lesion; two-way ANOVA; group *F*_(1,4)_ = 939.849, *p* < 0.001; time *F*_(13,52)_ = 64.358, *p* < 0.001; interaction *F*_(13,52)_ = 32.538, *p* < 0.001; Figure 4B). To further examine the role of small diameter afferent fibers in producing high acupoint conductance, we injected a specific C/Aδ-fiber blocker RTX into ulnar and median nerves 48–72 h prior to the IMH procedure. Unlike pretreatment of the nerves with saline (vehicle), pretreatment with RTX abolished the development of the high conductance at the acupoint in IMH rats (two-way ANOVA; group *F*_(1,4)_ = 398.84, *p* < 0.001; time *F*_(13,52)_ = 82.402, *p* < 0.001; interaction *F*_(13,52)_ = 25.643, *p* < 0.001; Figure 4C). Furthermore, Aδ/C-fiber blockade by RTX was confirmed by significantly less FITC IB4 tracer labeling in the DRG in RTX-treated rats (RTX/IB4) compared to in the vehicle group (vehicle/IB4, Figures 4D,E). Taken together, our results indicate that small diameter afferent fibers mediate the development of high conduction at acupoints.

### Production of High Conductance by SP and CGRP

**FIGURE 5:**
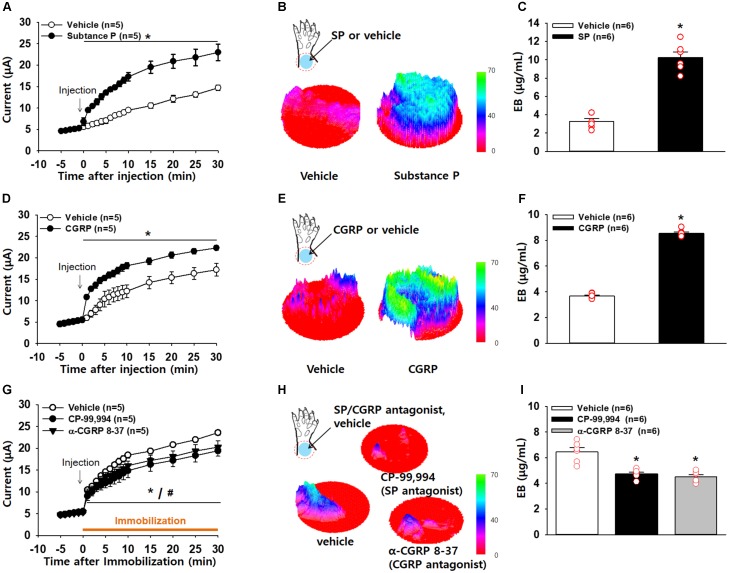
Increased conductance at acupoints by SP or CGRP. **(A–C)** Effect of intradermal substance P (SP) on electrical conductance in naïve rats. Injection of SP into the skin of the wrist acupoints significantly increased conductance compared to injection of vehicle **(A)**. *p* < 0.05 vs. vehicle. The three-dimensional (3D) images of EBD extravasation in the wrist skin in vehicle- or SP–treated rats **(B)** and the concentration of EBD in the wrist acupoints of vehicle- or SP-treated rats **(C)**. ^∗^*p* < 0.001 vs. vehicle. **(D–F)** Effect of intradermal calcitonin gene-related peptide (CGRP) on electrical conductance in naïve rats. Injection of CGRP into the skin of the wrist acupoints significantly increased conductance compared to injection of vehicle **(D)**. The 3D images of EBD extravasation in the wrist skin in vehicle- or CGRP-treated rats **(E)** and the concentration of EBD in the wrist acupoints of vehicle- or CGRP-treated rats **(F)**. ^∗^*p* < 0.001 vs. vehicle. **(G–I)** Effects of intradermal SP or CGRP antagonists on the development of high conductance in hypertensive rats. Injection of SP or CGRP antagonists into the skin of the wrist acupoints prevented the development of high conductance in IMH rats compared to injection of vehicle **(G)**. The 3D images of EBD in the wrist skin in vehicle- or SP or CGRP antagonist-treated rats **(H)** and the concentration of EBD in the wrist acupoints of vehicle- or SP or CGRP antagonist-treated rats **(I)**. ^∗^*p* = 0.003 vs. vehicle.

To explore whether the increased conductance at acupoints is associated with levels of SP and CGRP, we injected either SP or CGRP intradermally into the skin on the wrist and measured the skin conductance. An artificial increase in SP in the skin significantly increased conductance (two-way ANOVA; group *F*_(1,4)_ = 63.285, *p* = 0.001; time *F*_(19,73)_ = 117.661, *p* < 0.001; interaction *F*_(19,76)_ = 22.732, *p* < 0.001; Figure 5A). In another set of experiments, we determined the levels of plasma extravasation 2 h after the injection of vehicle (*n* = 6) or SP (*n* = 6) and found significantly higher levels of plasma extravasation in the wrist acupoints of SP-injected rats than in those of vehicle-injected rats, as shown in 3D skin images (Figure 5B) and the EBD concentration assessed by spectrophotometry (*t*-test, *p* < 0.01; Figure 5C). Such effects were replicated by injection of a CGRP agonist (two-way ANOVA; group *F*_(1,4)_ = 20.372, *p* = 0.011; time *F*_(19,76)_ = 122.989, *p* < 0.001; interaction *F*_(19,76)_ = 18.115, *p* < 0.001; Figures 5D–F).

To see if inhibition of SP or CGRP prevents the increase in conductance at acupoints, we injected either vehicle (saline), a SP antagonist CP-99,994 or a CGRP antagonist α-CGRP 8-37 into acupoints on the wrist prior to restraint and measured conductance up to 30 min after restraint. Saline-injected rats showed enhanced conductance at the wrist acupoints following restraint, which was significantly reduced by pretreatment with the SP or CGRP antagonist (two-way ANOVA; group *F*_(2,8)_ = 2.307, *p* = 0.162; time *F*_(19,76)_ = 277.166, *p* < 0.001; interaction *F*_(38,152)_ = 2.361, *p* < 0.001; Figure 5G). This decrease was further confirmed by 3D skin images (*t*-test; ^∗^*p* < 0.05, Figure 5H) and the EBD concentration in the skin (one-way ANOVA; ^∗^*p* < 0.05 vs. Vehicle, Figure 5I). Taken together, these results suggest that local release of SP and CGRP in acupoints leads to an increase in conductance by inducing vascular leakage.

### Increased Levels of SP and CGRP at Acupoints in IMH Rats

**FIGURE 6:**
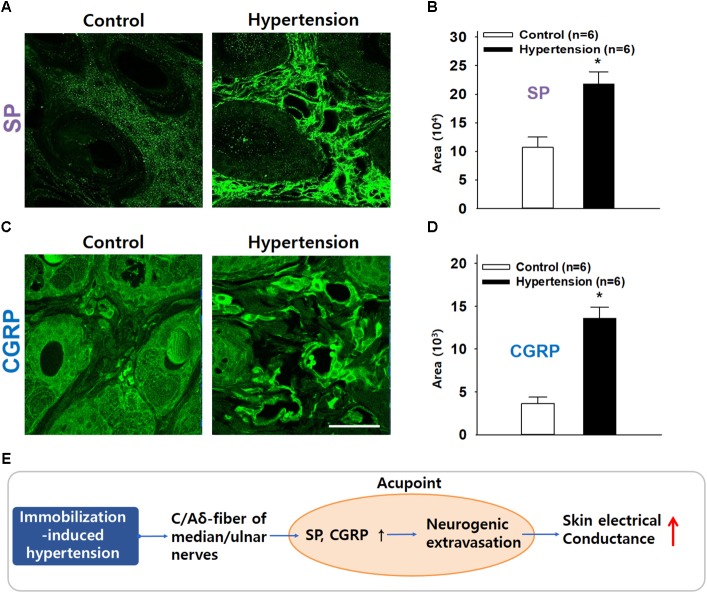
Increased immunofluorescence of SP or CGRP in the skin over acupoints. **(A,B)** Greater SP expression in the wrist acupoint in IMH rats than in control rats. **(C,D)** Greater CGRP expression in the wrist acupoint in IMH rats than in control rats. ^∗^*p* < 0.001 vs. control. Bar = 50 μm. **(E)** Overall hypothesis. A pathological condition of hypertension antidromically activates peripheral nerves, especially small diameter sensory afferents, which causes SP and CGRP release in the acupoints containing the activated sensory afferents and thus induces vasodilation and plasma extravasation. It leads to the development of high conductance at acupoints.

Finally, to confirm the increased expression of SP and CGRP at acupoints in IMH rats, we compared the expression of CGRP and SP in the skin on the wrist between normal (*n* = 6) and IMH rats (*n* = 6). Significantly greater SP (Figures. 6A,B) and CGRP (Figures 6C,D) fluorescence was found in the dermis of IMH rats than in that of control naïve rats (*t*-test; *p* < 0.001 in Figures 6B,D). Notably, the dermal blood vessels in IMH rats but not control naïve rats were predominantly enlarged (Figures 6A,C).

## Discussion

Our findings demonstrated that SP and CGRP released from afferents fibers increased electrical conductance at acupoints in IMH rats. In the IMH rat model, cutaneous neurogenic inflammation was found most frequently in the acupoints on the wrist. These acupoints revealed higher temperature and more plasma extravasation than the acupoints in naïve rats. Electrical conductance at the acupoints gradually increased with the development of hypertension but was blocked by surgical transection of the median and ulnar nerves or blockade of small diameter afferent fibers with RTX. Skin conductance and plasma extravasation were increased by intradermal injection of SP or CGRP in normal rats. In turn, inhibition of SP or CGRP by antagonists prevented the increase in both the conductance and plasma extravasation at acupoints in IMH rats. Levels of SP and CGRP were elevated in the dermis of acupoints in hypertensive rats. Our findings suggest that the local release of SP and CGRP induces vasodilation and plasma extravasation, resulting in accumulation of subskin tissue water content, thereby leading to an increase in electrical conductance at acupoints (Figure 6E).

While traditional acupoints have long been thought to be anatomically invisible, our recent study suggested that acupoints can be identified as neurogenic inflammatory spots (Neuro-Sp) on the skin, which are produced by activation of somatic afferents in abnormal conditions of visceral organs and can be visualized by intravenous injection of EBD (Kim et al., 2017). Consistent with our previous study (Kim et al., 2017), hypertensive rats revealed highly localized neurogenic inflammation (indicated by Neuro-Sp) in the dermatome of the forelimbs (Figure 1), which is innervated by the same spinal segments (C8-T2) that innervate the heart (Alles and Dom, 1985). Cutaneous neurogenic inflammation was observed most frequently in acupoints on the wrist, such as PC6, HT7, and PC7 (Figures 1D,E), which are commonly used in acupuncture clinics for cardiac disorders (Stux and Pomeranz, 2012). Furthermore, neurogenic inflammation tended to rapidly develop in acupoints after an IMH procedure and to be maintained during IMH, as shown by EBD staining and thermal recordings. Similar to our findings, a previous study reported that neurogenic inflammation appears in the skin of the abdomen, groin, lower back, and perineal areas several minutes after uterine inflammation with mustard oil, as assessed by EBD extravasation (Wesselmann and Lai, 1997). Cutaneous neurogenic inflammation is manifested as flare, vasodilation and increased local skin temperature (Wesselmann and Lai, 1997; Serra et al., 1998). In our thermal recordings and imaging, a rapid and long-lasting rise in temperature at acupoints was observed after the IMH procedure and was mimicked by intradermal injection of capsaicin, which produces cutaneous neurogenic inflammation (Lin et al., 1999). These results suggest that acupoints on the wrist displayed active neurogenic inflammation characterized by plasma extravasation in this rat model of hypertension.

Multiple studies have suggested that acupoints have higher conductance and lower impedance (resistance) than the surrounding skin (Reichmanis et al., 1975; Colbert et al., 2008, 2009), although this issue is currently controversial (Ahn et al., 2008; Wong, 2014). Reichmanis et al. (1975) reported that certain acupoints had significantly higher electrical conductance than nearby sites in healthy subjects. Colbert et al. (2008, 2009) recorded skin impedance at multiple acupoints simultaneously by using a fully automatic multichannel device and reported that several acupoints showed lower impedance than nearby sites in healthy subjects. The above studies are supported by our previous and current studies showing a high conductance of acupoints in IMH rats (Kim et al., 2017) (Figures 3D–F). However, others have also shown inconsistent results in the electrical conductance and impedance of acupoints. Pearson et al. (2007) observed that none of the three acupoints tested had lower skin impedance than the surrounding skin in healthy subjects. Kramer et al. (2009) reported that when skin impedance was measured in healthy subjects by using an array electrode of 64 channels, the majority of acupoints tested showed no changes in impedance, but some showed transient high or low impedance. A systematic review found that five out of nine studies showed a positive association between acupoints and low electrical impedance (Ahn et al., 2008). In these previous studies, a major problem is that almost all were conducted in healthy subjects and not in disease states, which may generate mixed results in electrodermal measurements at acupoints. As acupoints are generally accepted to reflect pathological states of the body and to become hypersensitive under pathological conditions (Stux and Pomeranz, 2012; Kim et al., 2017), pathological body conditions may cause considerable changes in skin conductance or impedance at acupoints. In support of this, our previous and present studies found that the conductance of acupoints significantly increased with the development of hypertension in rats, and such an increase was not seen in the same acupoints of control rats (Kim et al., 2017) (Figures 3D–F). In addition, the acupoints with high conductance in IMH rats but not naïve rats showed higher neurogenic inflammation and plasma extravasation than those of naïve rats (Figures 3G,H). Our hypothesis is further supported by another study showing that more significant changes in skin impedance at acupoint GB34 were observed in patients that had undergone surgery than in healthy subjects (Kramer et al., 2012). Importantly, previous studies point out that the precision of skin conductance measurements can be influenced by numerous factors such as skin dryness, skin thickness, size of the sensing electrode, pressure applied on the electrode, interelectrode distance, room temperature, and humidity (Ahn and Martinsen, 2007). In the present experiment, we developed a device that applied constant pressure on the electrode and performed the experiments under controlled environmental conditions, which might rule out the impact of the above factors. Therefore, the present study suggests that during diseases, the conductance at acupoints is abnormally high and that these electrical changes are associated with neurogenic inflammation and plasma extravasation.

In the somatic areas of referred pain from viscera, Neuro-Sp are generated by activation of small diameter sensory afferents (C/Aδ-fibers) in the dermatome convergent with visceral afferents (Wesselmann and Lai, 1997; Arendt-Nielsen et al., 2008). The sensory neurons are branched, with one projection leading to the internal organs and the other extending to the skin. The visceral inputs activate the viscerosomatic convergent neurons in the sensory pathway, and the neurons antidromically activate the branches, leading to the release of neuropeptides (e.g., SP and CGRP) from small diameter sensory fibers and subsequent neurogenic extravasation (Wesselmann and Lai, 1997; Arendt-Nielsen et al., 2008). Linkage of Neuro-Sp to internal organs was proven by our previous study showing convergent DRG neurons innervating both the heart and the Neuro-Sp (Kim et al., 2017). In the present study, the development of high conductance at acupoints in hypertensive rats was almost completely ablated by surgical lesions of the median and ulnar nerves (Figures 4A,B), suggesting that the afferent nerves mediate the high conductance at acupoints. Furthermore, pretreatment of the median and ulnar nerves with a specific C/Aδ-fiber blocker RTX prevented the development of high conductance at acupoints (Figure 4C), while RTX effectively blocked transmission of small sensory afferents in the median and ulnar nerves (Figures 4D,E), as reported previously (Suter et al., 2009). Taken together, these findings indicate that high conductance at acupoints is caused by antidromic activation of peripheral nerves, especially small diameter sensory afferents, in the dermatome associated with visceral disorders.

Activation of small diameter sensory afferents is known to induce the release of neuropeptides SP and CGRP into the periphery and lead to the development of neurogenic inflammation (Richardson and Vasko, 2002). SP as well as other tachykinins activates neurokinin receptors to increase microvascular permeability and edema formation, while CGRP acts on CGRP1 receptors to dilate arterioles (Schmelz and Petersen, 2001). The neuropeptides released by activated afferent fibers evoke neurogenic inflammation in the skin by activating vasodilation, axon reflex flare, and microvascular plasma extravasation (Wesselmann and Lai, 1997; Schmelz and Petersen, 2001). In the present study, intradermal injection of SP or CGRP increased both conductance and plasma extravasation in naïve rats, similar to the pattern observed in hypertensive rats (Figures 5A–F). In contrast, intradermal injection of SP or CGRP antagonists into acupoints prevented the development of high conductance and plasma extravasation at acupoints (Figures 5G–I). Moreover, increased levels of SP and CGRP were found in the acupoints of hypertensive rats. Thus, our findings suggest that SP and CGRP induce vasodilation and plasma extravasation to increase skin hydration, resulting in the development of high conductance at acupoints. Paradoxically, the locally released SP and CGRP into acupoints may in turn play a role in acupuncture effect. It has been suggested that active acupoints are associated with tissues where the sensory nerve endings are sensitized by neurogenic inflammatory mediators (Rong et al., 2013; He et al., 2017). Given that the sensitized sensory nerve endings are more sensitive to external stimuli than intact sensory nerves, we suggest that sensory nerve endings in acupoints would be sensitized by SP or CGRP released during neurogenic inflammation. Accordingly, stimulation of these sensitive acupoints would evoke the therapeutic effects of acupuncture by reaching physiological thresholds quickly, compared with stimulation of normal surrounding tissues including as sham or inactive acupoints.

A limitation of this study is that neurogenic extravasation of EBD was examined in only the skin over acupoints. Acupuncture needles often penetrate multiple layers including skin, subcutaneous tissue and muscles. As these layers may contain structures that respond to needling and produce acupuncture effects, future study will be needed to identify whether the neurogenic inflammatory processes also occur in the tissues below skin (i.e., subcutaneous tissue and muscles) and in turn are associated with acupuncture effects.

## Conclusion

The present study suggests a novel mechanism underlying the electrical properties of acupoints: the neuropeptides SP and CGRP produce high conductance at acupoints by causing neurogenic inflammation, plasma extravasation and accumulation of subskin water contents. This study would help solve some of controversial issues concerning electrical properties of acupoints.

## Author Contributions

HK designed the experiment, responsible for the overall direction of the project, and for edits to the manuscript. YF, D-HK, YR, SC, BL and CHY performed the experiments and analyzed the data. YF and HK drafted the manuscript.

## Conflict of Interest Statement

The authors declare that the research was conducted in the absence of any commercial or financial relationships that could be construed as a potential conflict of interest. The reviewer Y-HC and handling Editor declared their shared affiliation.
